# Primary and secondary prevention of cervical cancer during the COVID-19 pandemic in Sao Paulo State, Brazil

**DOI:** 10.1590/S1678-9946202668064

**Published:** 2026-09-18

**Authors:** Thainá Siqueira de Carvalho, Marcelo Alves Ferreira, João Carlos Geber, Sandra Lorente, Carlos Dias Maciel, Amaro Nunes Duarte-Neto

**Affiliations:** 1 Universidade de São Paulo, Faculdade de Medicina, Departamento de Fisiopatologia Experimental, São Paulo, São Paulo, Brazil; 2 Universidade de São Paulo, Faculdade de Medicina, Departamento de Biologia Celular, São Paulo, São Paulo, Brazil; 3 Universidade de São Paulo, Faculdade de Medicina, Departamento de Patologia, São Paulo, São Paulo, Brazil; 4 Instituto Adolfo Lutz, São Paulo, São Paulo, Brazil; 5 Universidade Estadual Paulista, Faculdade de Engenharia e Ciências, Departamento de Engenharia Elétrica, Guaratinguetá, São Paulo, Brazil

**Keywords:** Cervical cancer, Screening, Cytology, HPV, COVID-19

## Abstract

Cervical cancer remains an important public health problem. Prevention relies on vaccination against human papillomavirus (HPV) and Pap smears for early detection of precursor lesions. Both strategies should be implemented at appropriate intervals. However, the COVID-19 pandemic led to delays in preventive care, which may have negatively impacted cervical cancer (CC) indicators. Therefore, this study aims to assess the impact of the pandemic on the CC prevention program in Sao Paulo State, Brazil, using data from a public health database. An ecological study was conducted using quantitative data on HPV vaccination and Pap smears obtained from DATASUS between 2016 and 2021. Municipalities were grouped into clusters according to population size and their performance during 2020 and 2021 (increase or decrease in preventive services) and were compared according to socioeconomic variables. HPV vaccination rates increased in 2020, but this trend was not sustained in 2021. Regarding Pap smears, most municipalities experienced a decline in 2020, followed by an increase in 2021. Municipalities with the poorest socioeconomic indicators struggled to recover in 2021. The number of COVID-19 cases was not strongly associated with variations in preventive practices. CC prevention strategies were affected by the drop in HPV vaccination in 2021 and the postponement of routine screening during the most critical period of the pandemic, especially in the most vulnerable municipalities, which experienced a slower recovery. These findings suggest that the Brazilian healthcare system faced great challenges in both maintaining and restoring preventive care during a public health crisis.

## INTRODUCTION

The coronavirus disease 2019 (COVID-19) pandemic not only directly affected the lives of millions of people as an acute viral respiratory disease but also had an indirect negative impact on several healthcare sectors, as well as on society as a whole, including on the prevention and management of other diseases, such as cancer^1^. In response to the public health emergency, state governments imposed restrictions to limit virus transmission, such as suspension of in-person activities in schools and non-essential sectors^2^. Additionally, the Instituto Nacional de Cancer Jose Alencar Gomes da Silva (INCA) issued a statement on March 30, 2020, recommending that cancer screening practices be postponed until COVID-19 control measures were eased^3^.

Studies published since the most critical period of the pandemic have shown a decrease in the number of human papillomavirus (HPV) vaccine doses administered^4^ ^-^ ^6^ and in the number of preventive examinations performed during periods when the strictest public health measures were in place, such as Pap smear for cervical cancer (CC) prevention^1^ ^,^ ^7^ ^-^ ^9^. However, most of these studies have adopted a broad approach, overlooking regional disparities such as those observed within Brazilian states.

Although the natural history of CC is slow, delays in preventive practices during the pandemic have raised concerns about adverse long-term outcomes, such as a possible increase in the detection of high-grade lesions in the coming years. Since regional differences in access to healthcare services, even in states with a high Human Development Index (HDI), may reveal distinct scenarios regarding CC preventive practices and the impact of the pandemic on individual communities, this study aims to compare data on primary and secondary prevention actions across municipalities in Sao Paulo State, Brazil, between 2016 and 2021. Using information from the Departamento de Informatica do Sistema Unico de Saude (DATASUS - Department of Information Technology of the Unified Health System), differences in HPV vaccine doses administered and CC screening profiles were evaluated to assess the impact of the pandemic on preventive practices.

## MATERIALS AND METHODS

### Ethics

The retrospective nature of this study, which involved the analysis of government database information without identifying participants, eliminated the risk of breaches of confidentiality. Therefore, written informed consent was not required. This study was approved by the Research Ethics Committee of the Faculty of Medicine, University of Sao Paulo (Nº 5.900.270, CAAE 66896422.4.0000.0068). Patients and the public were not involved in any stage of this research.

### Study design and database

This ecological time-series study analyzed quantitative data on the administration of HPV vaccine doses and the performance of CC screening in Sao Paulo State, Brazil, between 2016 and 2021. Sao Paulo is the most populous state in the country, representing 22% of the Brazilian population^10^. The estimated incidence of CC was 7.58/100,000, corresponding to approximately 2,550 new cases in 2023, and the estimated mortality rate ranged from 2.61 to 4.45/100,000 in 2021^11^. Data were collected between November 2022 and March 2023, with an additional collection performed in October 2024.

### Data on primary prevention

Data on vaccinations administered by means of the Sistema Unico de Saude (SUS - Brazilian Unified Health System) are recorded in the National Immunization Program Information System (SI-PNI) and made available online on the DATASUS Tabnet platform. DATASUS is an online platform that provides information on health indicators, healthcare infrastructure, epidemiological data, and morbidity^12^. Vaccination coverage information available in SI-PNI are limited to the year 2015; therefore, for this study, we used the number of doses administered available in DATASUS, as these data are available up to 2022^13^ ^,^ ^14^. The following selection criteria were applied: State (Sao Paulo), Year/Month (2016-2021), Immunobiological (quadrivalent HPV vaccine, female and male), Age group (9-14 years), and Dose (first and second).

### Data on secondary prevention

Data on CC screening are recorded in either the Cancer Information System (SISCAN) or the Cervical Cancer Control Information System (SISCOLO). In Sao Paulo State, around 40% of Pap smears are registered in SISCAN, while the remainder are registered in SISCOLO, which is an offline system whose data are not publicly available on the national database^15^ ^,^ ^16^. Due to these issues in epidemiological data coverage, we used only quantitative data from the Sistema de Informacao Ambulatorial (SIA-SUS - SUS Outpatient Information System), which is used to process reimbursement for outpatient procedures performed within SUS and whose information is available on DATASUS^16^ ^,^ ^17^. Both types of establishments that use SISCAN or SISCOLO must record their procedures in SIA-SUS. Therefore, this source is less prone to loss of quantitative data, although it has the disadvantage of not providing epidemiological information^16^.

The quantitative data for Pap smears registered in SISCOLO were obtained from SIA-SUS using the code 0203010019 - Exame Citopatologico Cervico-Vaginal/Microflora (Cervical-Vaginal Cytological Examination/Microflora), with the following criteria: Pap smears by municipality of residence (Sao Paulo State), number of Pap smears approved for payment, year and month of payment processing, period from 2016 to 2021, and age range of 25 to 64 years. For the SISCAN quantitative data, SIA-SUS code 0203010086 - Exame Citopatologico Cervico Vaginal/Microflora-Rastreamento (Cervical-Vaginal Cytological Examination/Microflora Screening) was used, and the inclusion criteria were the same as those for the SISCOLO code. The total number of Pap smears was obtained by adding Pap smears approved for payment under the SISCOLO and SISCAN codes.

### Data on COVID-19

To verify whether there was a direct relationship between COVID-19 and CC prevention activities, data on COVID-19 cases made available on the epidemiological panel prepared by the Brazilian Ministry of Health were collected. These data are updated weekly by the State Health Departments (SES)^18^. The number of cases and deaths was selected using the following filters: Region (Southeast), State (Sao Paulo), Municipalities (all), and Year (2020; 2021).

### Data from UBS

To understand the profile of exam offerings during the pandemic, we analyzed the relationship between the number of Unidades Basicas de Saude (UBS - Basic Health Units) relative to the population and compared it with the number of exams performed relative to the population. Quantitative data from UBS were obtained from the Cadastro Nacional de Estabelecimentos de Saude (CNES - National Register of Health Establishments). UBS are responsible for administering vaccine doses and collecting cytological Pap smears, being essential for primary healthcare services offered by SUS^12^. Quantitative data from UBS were selected using the following filters: State (Sao Paulo), Municipalities (All), year/month of competency, period (2019 and 2020), and type of establishment (Health Care Center, UBS).

### Data from sociodemographic indicators

Due to the lack of full consolidation of census data at the time of collection, population projections available on the Sistema Estadual de Analise de Dados (SEADE - State Data Analysis System) were used^19^. Data on educational level were obtained from DATASUS^20^, using the following filters: Population aged 15 years or older; Educational level: women with completed primary education or higher and women with no education or incomplete primary education; State: Sao Paulo; Period: 2010; Sex: female; Municipalities: all. Data from the Municipal Human Development Index (MHDI) for income was obtained from DATASUS and Instituto Brasileiro de Geografia e Estatistica (IBGE - Brazilian Institute of Geography and Statistics)^21^ using the following filters: State: Sao Paulo; Municipalities: all; Indicator: 2010 Census MHDI. These data were collected in October 2024.

### Statistical analysis

The data were tabulated using Microsoft Excel, and analyses were conducted using R (version 4.4.1, The R Foundation). Comparisons of numerical variables, such as number of exams, vaccination rates, and number of inhabitants per UBS, were performed using the Kruskal-Wallis test after assessing distribution of data using the Kolmogorov-Smirnov test. The association between groups based on exam variation and sociodemographic strata was analyzed using the chi-square test. Prediction models were created for the rate of tests performed based on rates of COVID-19 cases, HPV vaccination, MHDI for income, and population size. Vaccination rate data were analyzed using principal component scores due to technical issues related to multicollinearity and variance inflation. The quality of these models was evaluated by the Akaike Information Criterion. In the 2020 model, the components used represented 67.91% of variance in the original data, while in 2021, the components represented 48.59%. Additionally, a spatial autocorrelation analysis was performed using the Moran Index. Results with p<0.05 were considered statistically significant for all tests.

## RESULTS

### Participants

Sao Paulo State has a population of 44,539,225 individuals distributed across 645 municipalities. The female population aged 5-14 years and the male population aged 10-14 years were estimated at 2,654,432 and 1,367,781, respectively, representing 6% and 3% of the state’s total population eligible to immunization. The female population aged 25-64 years was estimated at 13,147,750, representing 30% of the state’s total population eligible for screening^19^ ^,^ ^22^.

### Descriptive data

#### Primary prevention

**Figure 1:**
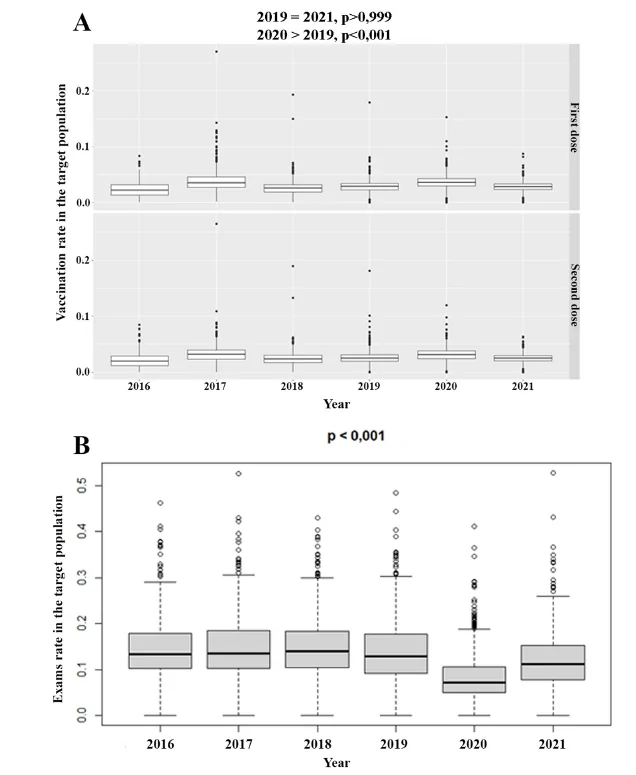
Panel with general data on preventive services: (A) HPV vaccination rate by dose. The HPV vaccination rate between 2016 and 2021 shows an increase in 2020 and a drop in 2021; (B) Pap smears performed between 2016 and 2021, presented proportionally to the female population. The number of tests performed remained stable until 2018, but a decrease was observed between 2019 and 2021, with p<0.001. This drop coincides with the start of the pandemic and the technical note issued by INCA recommending the postponement of cancer screening in the population without suspicion of malignancy until restrictive measures were relaxed. The graph presents the sum of the SISCAN and SISCOLO numbers available online at DATASUS, relative to the population of the municipalities.

Figure 1A shows HPV vaccination rates for the first and second doses in the target population of Sao Paulo State for 2016-2021. From 2016 to 2019, a trend toward stability was observed, with 2017 having the highest rate for both doses. From 2019 to 2020, a significant increase (p<0.001) was observed, beginning in April (Supplementary Figure S1), which was not maintained in 2021, when the rates returned to 2019 levels for both doses. Moreover, rates for the second dose were slightly lower than those for the first dose in all years.

#### Secondary prevention

Figure 1B shows the number of Pap smears performed in the female population between 2016 and 2021, presented proportionally to the female population. There was stability until 2018, with a slight drop in 2019, followed by a significant drop in 2020 and an increase in 2021, but the 2021 level did not reach that of before 2020. Variations over the years were statistically significant.

Table 1 shows clusters of municipalities constructed by comparing rates of Pap tests for the female population aged 25-64 across two consecutive periods: 2020 relative to 2019 and 2021 relative to 2020. Thus, each cluster aggregates annual trends of 2020 and 2021. Based on the potential year-over-year trends, four distinct clusters emerged: Increase-Increase, Increase-Decrease, Decrease-Increase, and Decrease-Decrease. The vast majority of municipalities belong to the Decrease-Increase group, which means that most municipalities in Sao Paulo experienced declines in 2020 and increases in 2021 compared to 2020.

**Table 1:** Clusters of municipalities grouped according to variations in the number of exams during the first two years of the pandemic. The number of exams in 2020 was compared to that in 2019, and the number of exams in 2021 was compared to that in 2020.

Cluster	Performance in 2020 compared to 2019	Performance in 2021 compared to 2020	Number of municipalities
Increase-Increase	Increase in 2020	Increase in 2021	41
Increase-Decrease	Increase in 2020	Decrease in 2021	16
Decrease-Increase	Decrease in 2020	Increase in 2021	525
Decrease-Decrease	Decrease in 2020	Decrease in 2021	63

**Figure 2:**
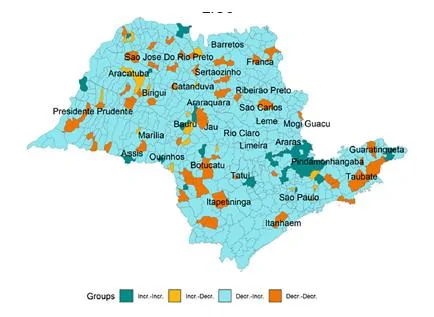
Spatial distribution of exam variation groups across the state. The names of municipalities with more than 100,000 inhabitants were included. Except for the Increase-Increase group, municipalities in the other groups are dispersed throughout the state. Although some municipalities in the Increase-Increase group are contiguous, the Moran Index showed no spatial autocorrelation among them (0.005, p=0.53).

The geographic distribution of the clusters is presented in Figure 2. Although there is contiguity among some municipalities in the Increase-Increase group, spatial autocorrelation was excluded based on the Moran Index (0.005, p=0.53). Conversely, the Increase-Decrease group had an increase in exams in 2020 and a decrease in 2021, showing the opposite behavior compared to most of the state. It is formed mainly by smaller municipalities located on the outskirts, except for Aracatuba and Bauru.

**Figure 3:**
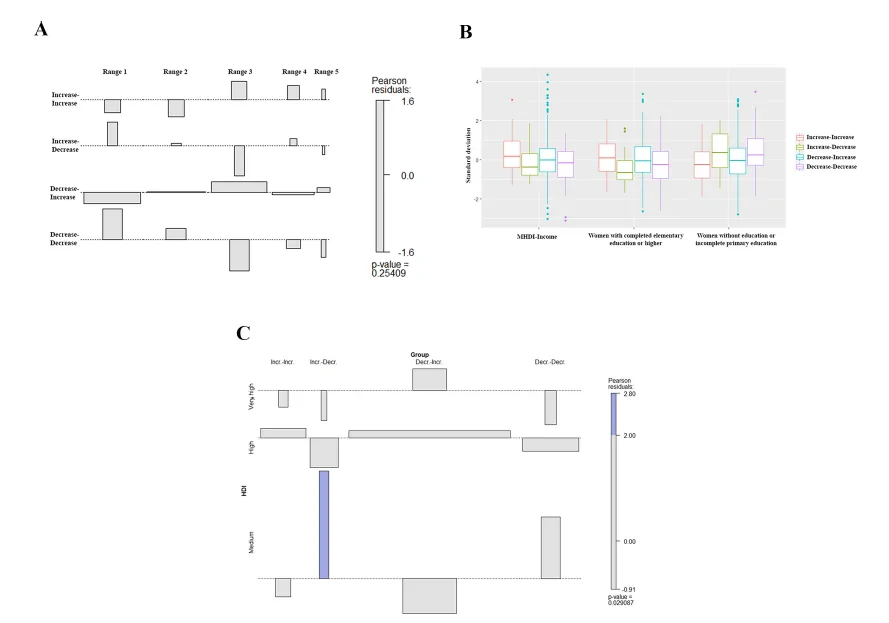
(A) Clusters of municipalities grouped by variation in exams and female population size ranges. The graph shows the Pearson residuals from a chi-square test. The height of the bars is proportional to the overrepresentation or underrepresentation of each comparison (exams variation clusters × female population ranges) in the sample. The bars above the lines (the Increase-Increase group in Ranges 3, 4, and 5; Increase-Decrease group in Ranges 1, 2, and 4; Decrease-Increase group in Ranges 2, 3, and 5; Decrease-Decrease group in Ranges 1 and 2) indicate greater representation of the exam variation group in the population range. Conversely, the lower bars and/or below the lines indicate sub-representation of the group within the population range (Increase-Increase group in Ranges 1 and 2; Increase-Decrease group in Ranges 3 and 5; Decrease-Increase group in Ranges 1 and 4; Decrease-Decrease group in Ranges 3, 4, and 5); (B) Differences between exam variation clusters in socioeconomic variables. The association between clusters and socioeconomic indicators was assessed using the chi-square test and presented as standard deviations for better visual comparison. The performance of municipalities varied according to their MHDI and education level; the Increase-Increase and Decrease-Increase clusters are formed by municipalities with the highest medians, i.e., the best socioeconomic indicators, and recovered in 2021, albeit partially. The Increase-Decrease and Decrease-Decrease clusters are formed mainly by municipalities with the lowest medians, i.e., lower education levels and lower MHDI, and were unable to increase the number of exams in 2021; (C) Classification of groups by Municipal Human Development Index (MHDI). The height of the bars is proportional to the overrepresentation or the underrepresentation of each comparison (exam variation clusters × MHDI classification) in the sample. Groups that experienced an increase in exams in 2021 were those classified as having high and very high MHDI, while those classified as having medium MHDI showed a decrease in 2021. The values ​​assigned to each HDI level are: Very High: 0.800-1.000; High: 0.700-0.799; Medium: 0.600-0.699.

Figure 3A shows these exam variation groups in a contingency association with the female population ranges of the municipalities. The size of each range is detailed in Supplementary Table S1. The bars above the lines indicate greater representation of the exam variation group in the population range, while the bars below the lines indicate lesser representation. Range 1 and Range 2 contain the smallest municipalities (Supplementary Table S1), and there is a greater representation of the Increase-Decrease and Decrease-Decrease groups in these ranges, i.e., municipalities that experienced a decrease in the number of exams in 2021. Ranges 3, 4, and 5 are heterogeneous and have greater representation of the Increase-Increase, Increase-Decrease, and Decrease-Increase groups. It was observed that the Increase-Increase group, which did not have its exam schedule affected by the pandemic, has greater representation in ranges of medium to large municipalities, while the Decrease-Increase group has municipalities represented in both small and large ranges. The Increase-Decrease group has greater representation mainly in small municipalities, but also in some municipalities in Range 4. These results show that the size of municipalities alone cannot explain variation in the number of exams.

Figure 3B shows differences in the MHDI income variables between the exam variation groups. Each boxplot represents a group, as indicated in the legend. The Increase-Increase and Decrease-Increase groups are made up of municipalities with highest income and highest education levels. The Increase-Decrease and Decrease-Decrease groups are made up of municipalities with the lowest incomes, incomplete elementary education, or no education. These data may show that higher income and education are factors that influenced the recovery of exams in 2021, albeit partially, since they were more frequent in groups with an increase in exams in 2021.

Figure 3C shows the association of groups with MHDI income. The bars above the lines indicate greater representation of the groups in the MHDI strata, while the bars below the lines indicate lesser representation. The groups that recovered in 2021 (Increase-Increase and Decrease-Increase) had greater representation in the high and very high MHDI strata. Conversely, groups that showed declines in 2021 (Increase-Decrease and Decrease-Decrease) had greater representation in the medium MHDI strata.

### Profile of exam offering

**Figure 4:**
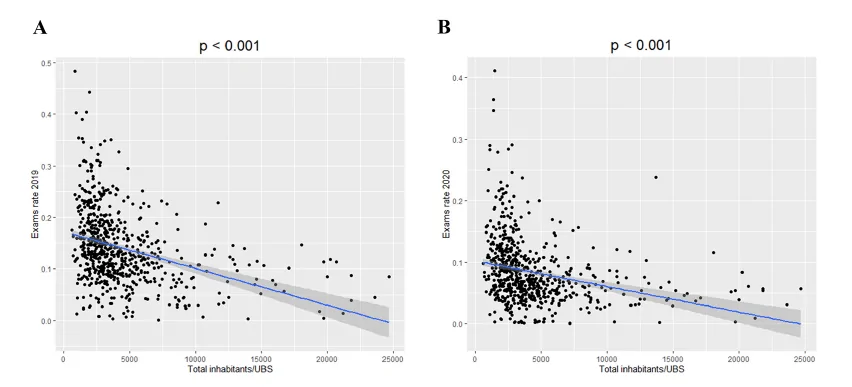
Number of inhabitants per UBS **× r**ate of exams by female population. The regression analysis showed a significant relationship (p<0.001) between a smaller number of inhabitants per UBS and higher rates of exams performed in both 2019 (A) and 2020 (B).

The analysis of the number of inhabitants per UBS showed that it follows the female population ranges, as shown in Supplementary Figure S2. The size of the female population ranges is the same as those defined for the analysis of the number of exams (Supplementary Table S1). Figure 4 shows the number of inhabitants per UBS according to the rate of exams performed in the female population in 2019 and 2020. Despite the proportionality between number of inhabitants per UBS and population size (Supplementary Figure S2), the rate of exams performed does not follow this pattern, as evidenced by the negative regression line, showing that as the number of inhabitants per UBS increases, the rate of exams decreases. This pattern remained similar before and during the pandemic.

### Impact of COVID-19 on prevention services

**Figure 5:**
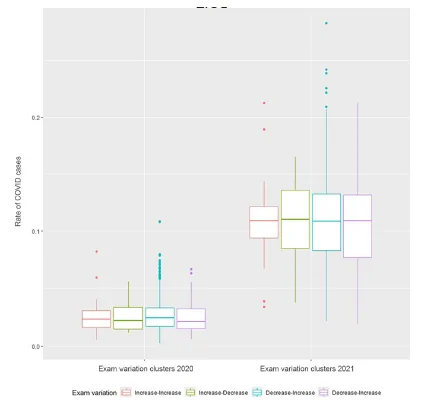
COVID-19 cases in 2020 and 2021 in relation to the total population of Sao Paulo State **× exam** variation clusters. The exam variation groups showed no significant differences in COVID-19 case rates.

The analysis of COVID-19 cases proportional to the total population in 2020 and 2021, stratified by exam variation groups (Figure 5), shows no differences in COVID-19 rates among municipalities in the four groups in either year. These data may suggest that the volume of cases in the municipalities was not strongly associated with the decrease in tests during the pandemic, although there was a weak association with the rate of cases in regression models that controlled other factors, including population size. Supplementary Table S2 shows the multiple regression analysis between the variation rate of Pap smears with COVID-19 rate, MHDI of income, population size, and HPV vaccination. Despite statistical significance, the association was weak: the coefficient of determination was 15% for 2020 and 19% for 2021, with p<0.000001.

## DISCUSSION

This study shows, by analyzing data from DATASUS, that the primary prevention strategy for CC had a general increase during the critical phase of the COVID-19 pandemic in 2020 and returned to 2019 levels in 2021. The secondary prevention strategy experienced substantial declines in 2020 and only a partial recovery in 2021. Socioeconomic variables influenced CC screening recovery, but the declines could not be strongly associated with COVID-19 case rates.

Despite social distancing measures, our study indicated an increase in vaccination rates, notably beginning in April 2020, followed by a decline in 2021. Similar findings were reported in an analysis of the SI-PNI database by Silva*et al*.^4^, who observed an upward trend in the median number of doses administered beginning in May 2020. These findings potentially reflect guidelines issued by the Ministry of Health to resume the children vaccination schedule on April 16, 2020, which had been postponed to prioritize older adults influenza vaccination campaign. The 2021 declines could be attributed to the greater allocation of professionals and resources to the care of patients with respiratory syndromes, as well as the prioritization of COVID-19 vaccination campaigns, which began that year^24^ ^,^ ^25^. In Germany, HPV vaccination coverage among girls aged 9-14 dropped by 7.4% in 2020 and decreased even further to 21.2% in 2021^26^. In addition to the challenges posed by the pandemic, HPV immunization remains a global challenge, with declines being reported in low- and middle-income countries, including Brazil^27^ ^,^ ^28^. These findings highlight the need to enhance public confidence and strengthen childhood vaccination programs, even in times of crisis.

Our analysis of secondary prevention of CC corroborates the literature published since the beginning of the pandemic. According to SISCAN data analyzed by INCA, a sharp decrease in the number of Pap smears performed between 2019 and 2020 was observed in all Brazilian regions. Data for Sao Paulo State were estimated at 672,626 Pap smears in 2019 and 412,550 in 2020, representing a decrease of 260,076 Pap smears^9^. Duarte *et al*.^29^ found similar results for Sao Paulo, based on data from the Sistema de Informacoes Hospitalares (SIH - Hospital Information System) and the Autorizacao de Internacao Hospitalar (AIH - Hospital Admission Authorization). Furthermore, the authors found a decrease in procedures related to the treatment of CC precursor lesions and invasive neoplasia, which they attributed to the pandemic, suggesting that it may have also affected the population with suspected malignancy^29^. Another study, which analyzed data from 55 municipalities served by Fundacao Oncocentro de Sao Paulo (FOSP - Sao Paulo Cancer Foundation), also found a decrease in Pap smear numbers in 2020, followed by a recovery in 2021 to levels comparable to those of 2019^8^. The difference between their data and ours may be due to the fact that the authors limited their analysis to Pap smears performed by FOSP, which served 55 municipalities, while our analysis covered all 645 municipalities in the state. Some reasons that may explain this negative impact are the provisional postponement of cancer screening recommended by INCA^3^ and the need to reassign healthcare professionals to COVID-19 management^8^, which may have reduced the availability of preventive services. Additionally, fear of contamination may have discouraged women from seeking primary care^8^ ^,^ ^29^.

The profile of exam offering shows an accumulation of tests in places with fewer inhabitants per UBSs, which may be explained by the possibility of overestimation of exams performed proportionally to the female population. According to Brazilian guidelines, the Pap smear should be repeated annually until two consecutive negative results are obtained, and then every three years as long as the result is negative. For positive results or unsatisfactory samples, the repeat interval should be shorter^30^. As this study has an ecological design and therefore used a non-individualized database, it is possible that the same woman was tested more than once in the same year, while others may not have been tested or may have been tested outside the recommended interval. As a result, some underestimation of the number of women who were actually screened in each municipality is to be expected. Moreover, the opportunistic profile of screening, in which women seek healthcare services for reasons not necessarily related to prevention, and the greater use of private health services reinforce this scenario^31^. Furthermore, our analysis did not distinguish between Pap smears performed for screening, follow-up of positive cases, or repeat testing of unsatisfactory samples, which increases the likelihood of some overestimation of the number of exams.

The association between the number of Pap smears and sociodemographic variables showed that municipalities with the lowest levels of education and medium MHDI of income were unable to resume routine prevention in the target population, although regression analysis demonstrated a weak relationship with MHDI. According to the latest World Health Organization (WHO) data, countries with medium/low HDI have higher incidence and mortality rates from CC than those with high/very high HDI^32^. This highlights that, despite the reduction in CC incidence and mortality rates in recent decades, socioeconomic disparities remain obstacles to the elimination of this neoplasm as a public health problem^33^ ^,^ ^34^. The analysis by Buskwofie *et al*.^35^ in the United States demonstrated that less urbanized regions had lower rates of preventive examinations and higher incidence and mortality rates. In Maranhao State, Brazil, the chance of detecting CC precursor lesions at advanced stages was higher among women living in municipalities with low/very low HDI^36^. In Sao Paulo, disparities in the Social Responsibility Index (IRS) showed that early detection of CC is more likely under favorable social conditions^37^. Inefficient organization of screening programs, low educational level of the population, long travel times to healthcare units, transportation costs, time away from work, and unavailability of physicians are some of the barriers faced by these regions^34^ ^,^ ^35^, which may suggest a greater impact of the pandemic on more vulnerable regions.

Regarding COVID-19 data, our analysis did not show a strong influence of COVID-19 case rates on Pap smear rates. Despite this, we hypothesize that the later progression of the pandemic to inland municipalities may have indirectly impacted the Increase-Decrease group^24^ ^,^ ^38^, along with other variables not identified by our study. Since the Increase-Decrease group is mostly composed of municipalities in the regions of Aracatuba (2), Barretos (1), Bauru (4), Campinas (1), Greater Sao Paulo Area (2), Marilia (2), Presidente Prudente (1), and Sao Jose do Rio Preto (3), these municipalities may have maintained their preventive routines until the widespread dissemination of the virus. Studies that analyzed the flow of COVID-19 cases in the state showed that Aracatuba and Sao Jose do Rio Preto experienced a peak of severe acute respiratory disease in 202^38^. Moreover, Bauru and Aracatuba recorded the highest relative risks for COVID-19 from the end of 2020^24^, which may indicate that the difficulties faced in managing patients negatively impacted CC prevention strategies in the later stages of the pandemic.

Despite the significance of the associations between variations in Pap smear rates and COVID-19 case rates, MHDI of income, HPV vaccination rates, and population size, the analysis showed a weak relation, which may indicate that these factors were not sufficient to explain variations in Pap smear rates. Especially regarding COVID-19 data, the case rate was not strongly associated with variations in Pap smear rates, which could suggest that the overload of healthcare services was not the only reason for these variations. Other variables, such as the fear generated by stay-at-home measures, may have had a greater impact^1^ ^,^ ^39^ ^,^ ^40^.

### Limitations

The limitations of this study were mainly related to the quality and integrity of DATASUS data, as the collection of CC screening information is still undergoing a transition from SISCOLO to SISCAN. Due to this transition, SISCAN coverage is heterogeneous among Brazilian regions and municipalities^15^ ^,^ ^40^. Another limitation is the use of population projections instead of census data, which were not consolidated at the time of data collection and may have generated, to some extent, underestimation or overestimation in proportionality calculations. Furthermore, the use of a secondary data source made our results more susceptible to the influence of confounding variables that we were unable to identify and control, especially in the multiple regression analysis.

The strengths of this study include the stratification of the analysis by clusters of municipalities according to variations in their examination profiles, which made it possible to highlight some behaviors that were masked in analyses considering the entire state as a single cluster. This analysis is relevant because municipalities in Sao Paulo differ in population size and socioeconomic indicators, which may influence the evaluation of some variables. Moreover, the analysis proportional to population size enabled a better understanding of the behavior of municipalities according to their population size. Additionally, the analysis of a four-year pre-pandemic period enabled a better delineation of baseline primary and secondary prevention practices before COVID-19. Another point worth highlighting was the use of financial data from SIA-SUS, rather than only epidemiological data from SISCAN, which made it possible to include municipalities with lower coverage of the latter system in the analysis.

## CONCLUSION

Our study showed that the pandemic negatively affected preventive services in most municipalities in Sao Paulo, highlighting difficulties faced by the public healthcare system in maintaining preventive measures. Primary prevention increased in 2020, which was not maintained in 2021 and municipalities with better socioeconomic indicators achieved a partial recovery in secondary prevention in 2021. The analysis by groups showed different patterns in the management of CC prevention services. It remains to be seen whether there will be a full recovery, or even an increase, in preventive practices post-pandemic.

## SUPPLEMENTARY MATERIAL

SUPPLEMENTARY MATERIAL

## Data Availability

The complete anonymized dataset supporting the findings of this study is available from https://doi.org/10.48331/SCIELODATA.LQGYBN
