# Supplementary material for: Primary and secondary prevention of cervical cancer during the COVID-19 pandemic in Sao Paulo State, Brazil
Source: Rev Inst Med Trop Sao Paulo. 2026 Sep 18;68:e64. doi: 10.1590/S1678-9946202668064 (PMC13588560; doi:10.1590/S1678-9946202668064)
Supplement: Supplementary file 1 [file 1678-9946-rimtsp-68-S1678-9946202668064-md1.pdf]

REVISTA DO INSTITUTO DE MEDICINA TROPICAL DE SÃO PAULO

JOURNAL OF THE SÃO PAULO INSTITUTE OF TROPICAL MEDICINE

SUPPLEMENTARY MATERIAL

https://doi.org/10.48331/SCIELODATA.LQGYBN

Primary and secondary prevention of cervical cancer during the COVID-19 pandemic in Sao Paulo State, Brazil

Thainá Siqueira de Carvalho [1], Marcelo Alves Ferreira [2], João Carlos Geber Júnior [3], Sandra Lorente [4], Carlos Dias Maciel [5*], Amaro Nunes Duarte-Neto [3*]

[1]Universidade de São Paulo, Faculdade de Medicina, Departamento de Fisiopatologia Experimental, São Paulo, São Paulo, Brazil

[2]Universidade de São Paulo, Faculdade de Medicina, Departamento de Biologia Celular, São Paulo, São Paulo, Brazil

[3]Universidade de São Paulo, Faculdade de Medicina, Departamento de Patologia, São Paulo, São Paulo, Brazil

[4]Instituto Adolfo Lutz, São Paulo, São Paulo, Brazil

[5]Universidade Estadual Paulista, Faculdade de Engenharia e Ciências, Departamento de Engenharia Elétrica, Guaratinguetá, São Paulo, Brazil

*These authors are senior authors of the article

Correspondence to: Amaro Nunes Duarte-Neto
Universidade de São Paulo, Faculdade de Medicina, Departamento de Patologia, Av. Dr. Arnaldo, 455, sala 1149, CEP 01246-903, São Paulo, SP, Brazil
E-mail: amaro.ndneto@hc.fm.usp.br

Received: 8 February 2026
Accepted: 23 June 2026

Editor: Lorenza Nogueira Campos Dezanet

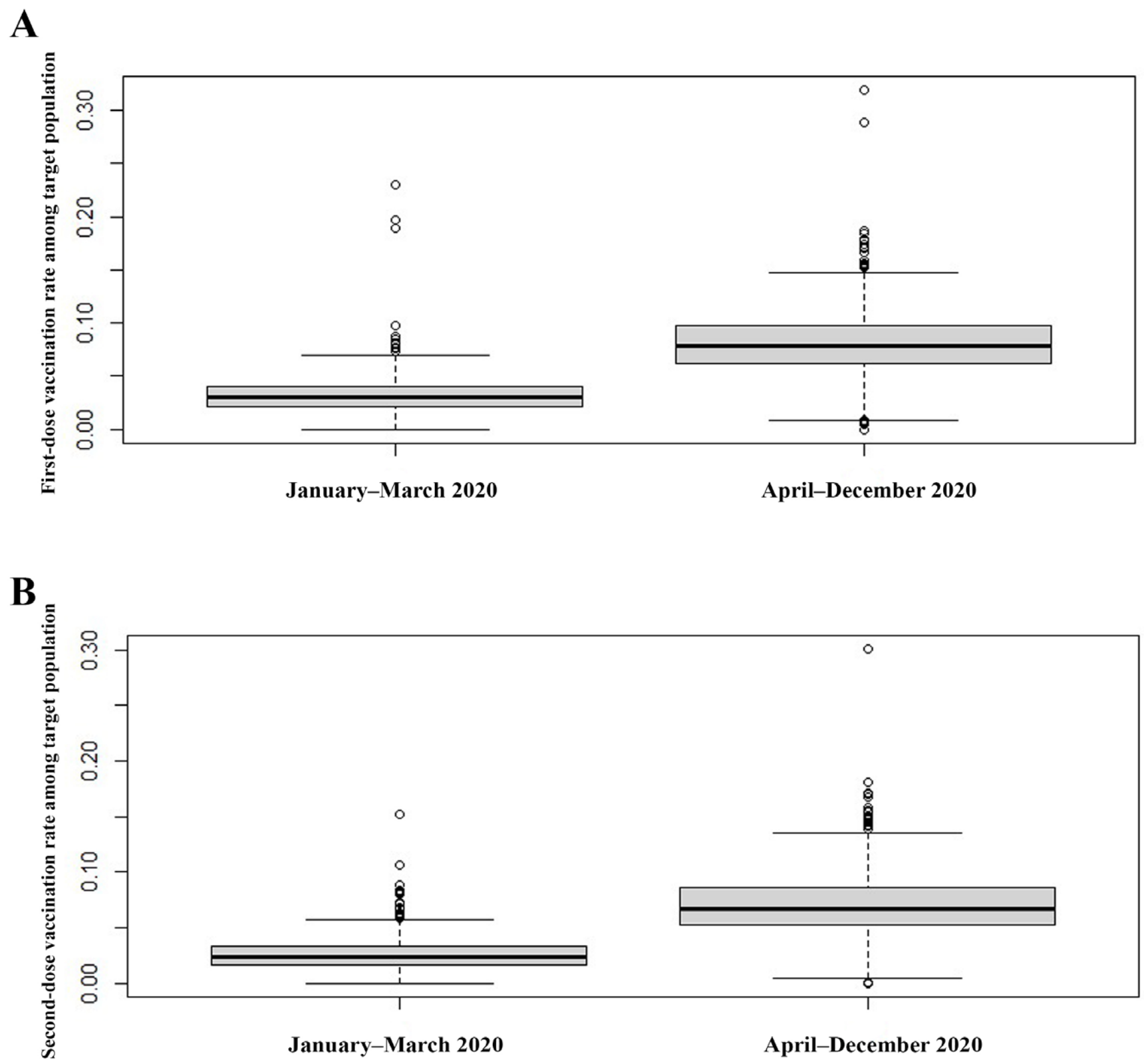

Supplementary Figure S1 - Vaccination rates before and after the implementation of social distancing measures: (A) First-dose vaccination rate among the target population initially between January and March 2020 and between April and December 2020. The comparison, performed using the chi-square test, showed an increasing trend after April. Similar findings were obtained for the second dose (B).

This is an open-access article distributed under the terms of the Creative Commons Attribution License.

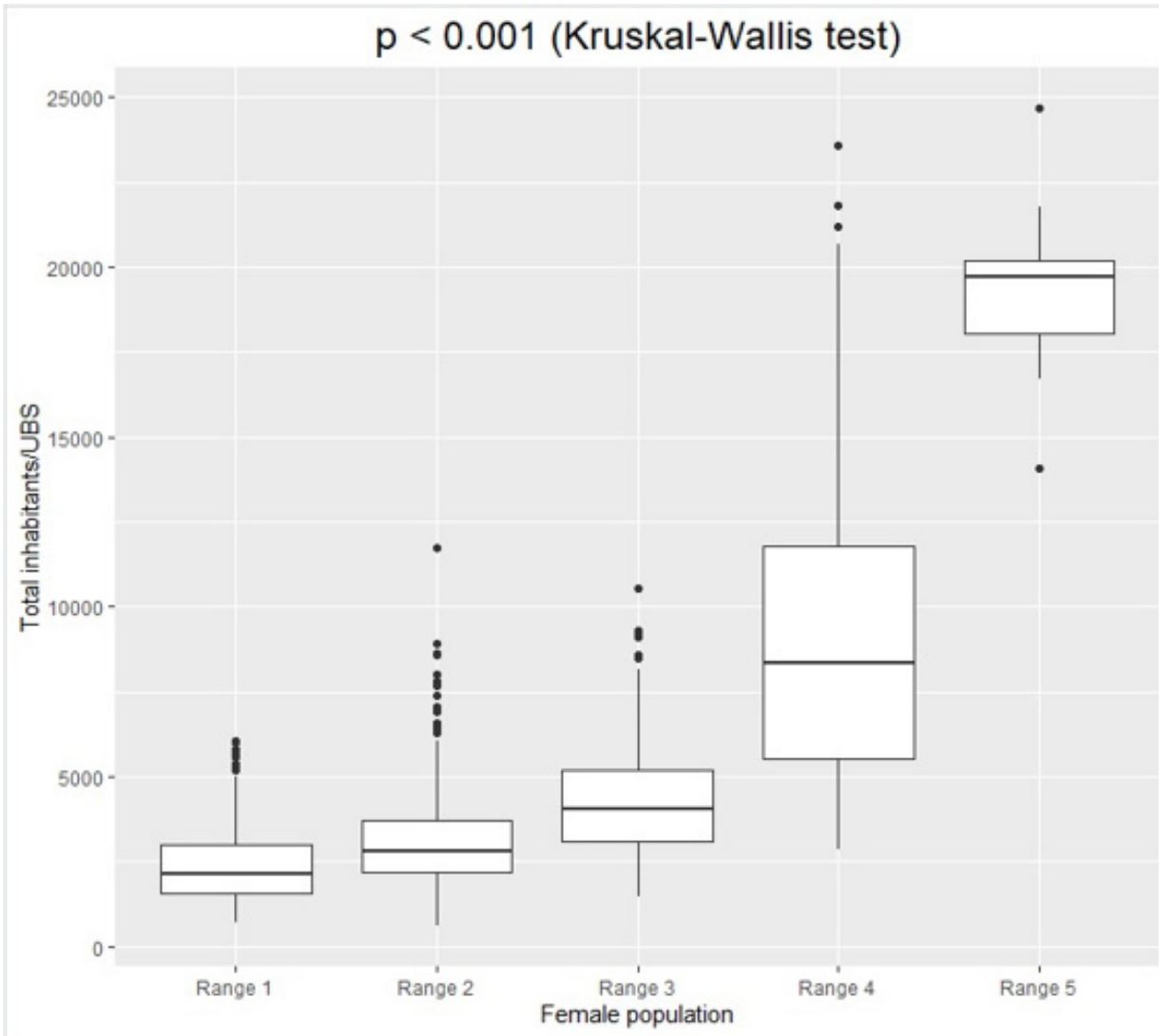

Supplementary Figure S2 - Number of inhabitants per UBS × female population size ranges. This comparison shows that the number of inhabitants per UBS is significantly (p<0.001) proportional to the population ranges, which are defined according to the female population size.

Supplementary Table S1 - Female population ranges.

Range	Female population
1	Up to 2,000
2	Between 2,001 and 6,000
3	Between 6,001 and 24,000
4	Between 24,001 and 220,000
5	More than 220,000

Supplementary Table S2 - Regression models for Pap tests rates in 2020 and 2021. The variables included in the models were those that maximized their quality according to the Akaike Information Criterion. Ranges: female population ranges as described in Table 1. Vaccination scores = scores obtained from the Principal Component Analysis of vaccination rates. *p*-value = significant associations are marked with an asterisk (*).

Variable	Estimate (Pap tests in 2020)	p-value (Pap tests in 2020)	Estimate (Pap tests in 2021)	*p*-value (Pap tests in 2021)
Intercept	0.934	<0.0001*	0.147	<0.0001*
COVID-19 case rates in 2020	+0.481	0.0004*	+0.347	0.031*
Range 2	–0.020	<0.0001*	–0.031	<0.0001*
Range 3	–0.037	<0.0001*	–0.050	<0.0001*
Range 4	–0.047	<0.0001*	–0.073	<0.0001*
Range 5	–0.047	0.004*	–0.091	<0.0001*
Municipalities with medium MDHI	-	-	–0.024	0.00226*
Municipalities with very high MDHI	-	-	+0.010	0.452
Vaccination score 1	+0.095	0.148	-	-
Vaccination score 2	+0.131	0.144	+0.163	0.118
Vaccination score 3	+0.179	0.992	+0.326	0.009*
Vaccination score 5	-	-	+0.410	0.043*
Vaccination score 6	-	-	–0.318	0.130
Vaccination score 10	+ 0.696	0.06	+ 0.973	0.026*
Vaccination score 11	+ 0.988	0.013*	+ 0.795	0.087
